# Measuring right ventricular volume and ejection fraction with Simpson's method: which MRI axis is best? Comparison with a "gold standard"

**DOI:** 10.1186/1532-429X-11-S1-O98

**Published:** 2009-01-28

**Authors:** Shawn Haji-Momenian, Kevin J Chang, David J Grand, Florence H Sheehan, Michael K Atalay

**Affiliations:** 1 Brown University, Providence, RI USA; 2 University of Washington, Seattle, WA USA

**Keywords:** Right Ventricular, Short Axis, Subdivision Surface, Right Ventricular Volume, Experienced Reviewer

## Purpose

Because of its complex morphology, accurate and reliable quantification of right ventricular (RV) volume and function using MRI is challenging. This study had two aims: (1) to determine the interobserver reliability of RV volume and ejection fraction (EF%) calculated using Simpson's method of slice summation applied to data acquired in three different orientations: short axis (SA), transaxial (TA), and parallel to the horizontal long axis (pHLA); and (2) to determine how RV volume and EF% by each of the three orientations compared with values obtained using a validated "gold-standard" method with 3-D reconstructions (3DR).

## Materials and methods

Twenty-three consecutive, consented patients referred for cardiac MRI were included in the study (10 males, 13 females; ave. age 43 ± 19 yrs; ave. ht: 66 ± 4 in; ave. wt: 177 ± 51 lbs). Steady-state free precession was used to generate stacked, bright-blood cine loops in 3 separate, randomly ordered orientations: SA, TA, and pHLA. Slice thickness was 8 mm and slice separation 2 mm. Using Simpson's method and pre-determined end-diastolic (ED) and end-systolic (ES) time points, three experienced reviewers independently measured RV ED and ES volumes and, in turn, EF% for each of the orientations. Volumes and EF% were also calculated using a 3DR technique based on the piecewise smooth subdivision surface method [1, 2], employing data from multiple orientations. Intraclass correlation was used to compare data from different observers. Paired t-test analysis was used to compare volumes and EF%.

## Results

Interrater reliability (IRR) of RV ED and ES volumes and EF% was determined for each of the axes. For the SA, IRR for all readers was 0.92, 0.87, and 0.33, respectively; for TA, 0.95, 0.90, and 0.71; for pHLA, 0.83, 0.91, and 0.67. (A higher ratio indicates greater reliability). For comparison, the same parameters were also determined for LV ED and ES volumes, and EF% measured using the SA: 0.98, 0.96, and 0.84.

**Figure 1:**
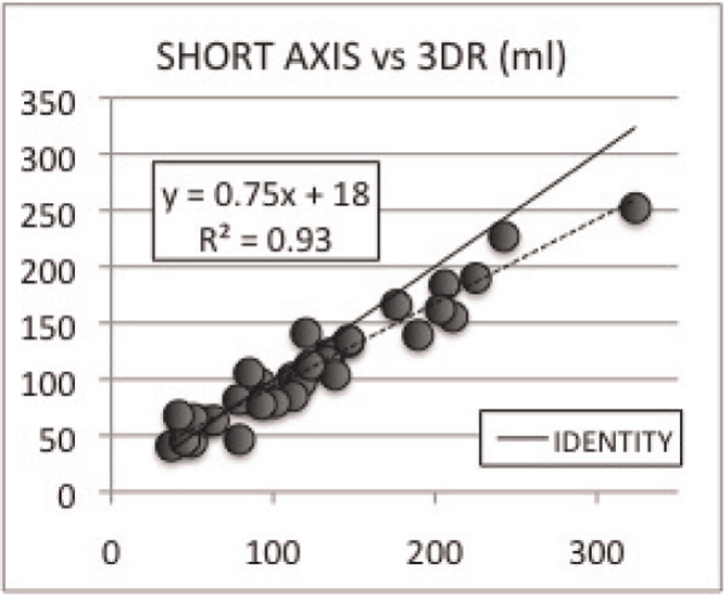
**Short axis vs 3DR (ml)**.

**Figure 2:**
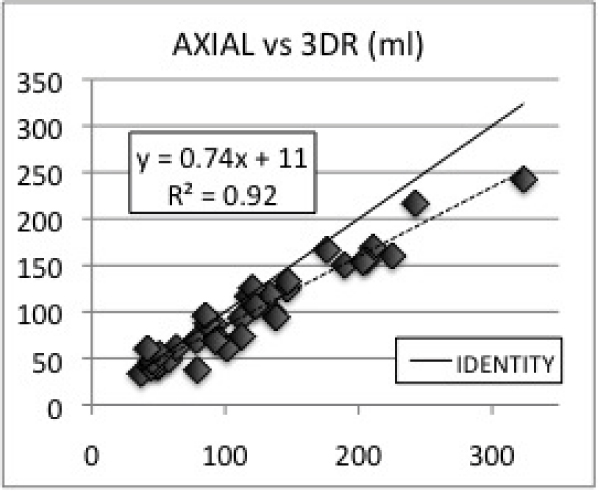
**Axial vs 3DR (ml)**.

**Figure 3:**
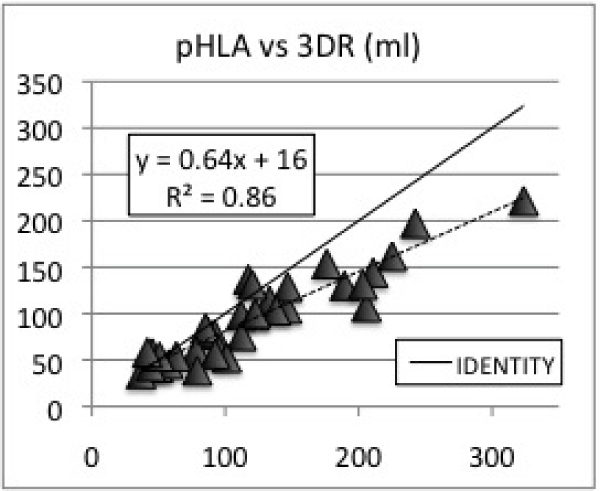
**pHLA vs 3DR (ml)**.

A wide range of RV volumes (37–323 cc) and EF%s (30–67%) were observed. Average EDV volumes for 3DR, SA, TA, & pHLA were: 161 ± 60 cc, 140 ± 45 cc, 136 ± 44 cc, 126 ± 37 cc. Average ESV volumes for 3DR, SA, TA, & pHLA were: 80 ± 44 cc, 76 ± 35, 65 ± 31 cc, 62 ± 30 cc. All ED and ES volumes were underestimated using Simpson's method (p < .01). SA, TA, and pHLA volumes were linearly correlated with 3DR volumes with R values 0.96, 0.96, and 0.93 (See Figures 1, 2, 3). Correlations for EDVs were slightly better than those for ESVs for SA and TA orientations. Average EF%s for 3DR, SA, TA, & pHLA were: 53 ± 9%, 47% ± 8% (p < .05), 53 ± 7% (P = NS), & 52 ± 9% (P = NS). SA, TA, and pHLA EF%s were linearly correlated with 3DR EF%s with R values 0.66, 0.57, and 0.65.

## Conclusion

Reliability of RV ED and ES volume measurements is comparable for all three axes evaluated. However, the reliability of the EF% is best on TA imaging. Moreover, although TA offers slightly worse EF% correlation with 3DR, our data suggest that for consistency – if Simpson's method is used for RV volume and EF% quantification – the TA axis is preferred.
